# CArdiac MagnEtic resonance assessment of bi-Atrial fibrosis in secundum atrial septal defects patients: CAMERA-ASD study^[Author-notes jeab188-FM1]^

**DOI:** 10.1093/ehjci/jeab188

**Published:** 2021-09-26

**Authors:** Louisa O’Neill, Iain Sim, Daniel O’Hare, John Whitaker, Rahul K Mukherjee, Orod Razeghi, Steven Niederer, Matthew Wright, Amedeo Chiribiri, Alessandra Frigiola, Mark D O’Neill, Steven E Williams

**Affiliations:** Division of Imaging Sciences and Biomedical Engineering, King’s College London, 4th Floor North, Wing, St. Thomas’, Hospital, London SE1 7EH, UK; Division of Imaging Sciences and Biomedical Engineering, King’s College London, 4th Floor North, Wing, St. Thomas’, Hospital, London SE1 7EH, UK; Division of Imaging Sciences and Biomedical Engineering, King’s College London, 4th Floor North, Wing, St. Thomas’, Hospital, London SE1 7EH, UK; Division of Imaging Sciences and Biomedical Engineering, King’s College London, 4th Floor North, Wing, St. Thomas’, Hospital, London SE1 7EH, UK; Division of Imaging Sciences and Biomedical Engineering, King’s College London, 4th Floor North, Wing, St. Thomas’, Hospital, London SE1 7EH, UK; Division of Imaging Sciences and Biomedical Engineering, King’s College London, 4th Floor North, Wing, St. Thomas’, Hospital, London SE1 7EH, UK; Division of Imaging Sciences and Biomedical Engineering, King’s College London, 4th Floor North, Wing, St. Thomas’, Hospital, London SE1 7EH, UK; Division of Imaging Sciences and Biomedical Engineering, King’s College London, 4th Floor North, Wing, St. Thomas’, Hospital, London SE1 7EH, UK; Division of Imaging Sciences and Biomedical Engineering, King’s College London, 4th Floor North, Wing, St. Thomas’, Hospital, London SE1 7EH, UK; Department of Cardiology, Guy’s and St Thomas’ NHS Foundation Trust, UK; Division of Imaging Sciences and Biomedical Engineering, King’s College London, 4th Floor North, Wing, St. Thomas’, Hospital, London SE1 7EH, UK; Division of Imaging Sciences and Biomedical Engineering, King’s College London, 4th Floor North, Wing, St. Thomas’, Hospital, London SE1 7EH, UK

**Keywords:** atrial septal defect, right atrium, atrial arrhythmias, cardiac MRI, late gadolinium enhancement

## Abstract

**Aims:**

Atrial septal defects (ASD) are associated with atrial arrhythmias, but the arrhythmia substrate in these patients is poorly defined. We hypothesized that bi-atrial fibrosis is present and that right atrial fibrosis is associated with atrial arrhythmias in ASD patients. We aimed to evaluate the extent of bi-atrial fibrosis in ASD patients and to investigate the relationships between bi-atrial fibrosis, atrial arrhythmias, shunt fraction, and age.

**Methods and results:**

Patients with uncorrected secundum ASDs (*n* = 36; 50.4 ± 13.6 years) underwent cardiac magnetic resonance imaging with atrial late gadolinium enhancement. Comparison was made to non-congenital heart disease patients (*n* = 36; 60.3 ± 10.5 years) with paroxysmal atrial fibrillation (AF). Cardiac magnetic resonance parameters associated with atrial arrhythmias were identified and the relationship between bi-atrial structure, age, and shunt fraction studied. Bi-atrial fibrosis burden was greater in ASD patients than paroxysmal AF patients (20.7 ± 14% vs. 10.1 ± 8.6% and 14.8 ± 8.5% vs. 8.6 ± 6.1% for right and left atria respectively, *P* = 0.001 for both). In ASD patients, right atrial fibrosis burden was greater in those with than without atrial arrhythmias (33.4 ± 18.7% vs. 16.8 ± 10.3%, *P* = 0.034). On receiver operating characteristic analysis, a right atrial fibrosis burden of 32% had a 92% specificity and 71% sensitivity for predicting the presence of atrial arrhythmias. Neither age nor shunt fraction was associated with bi-atrial fibrosis burden.

**Conclusion:**

Bi-atrial fibrosis burden is greater in ASD patients than non-congenital heart disease patients with paroxysmal AF. Right atrial fibrosis is associated with the presence of atrial arrhythmias in ASD patients. These findings highlight the importance of right atrial fibrosis to atrial arrhythmogenesis in ASD patients.

## Introduction

The prevalence of atrial arrhythmias in patients with secundum atrial septal defects (ASDs) is significantly greater than in the general population without ASDs,^1^ but little is known about the unique bi-atrial arrhythmia substrate in these patients. Although significant atrial dilatation may occur as a consequence of prolonged exposure to a left-to-right shunt,^2^ this tends to reverse after ASD closure although arrhythmia risk remains elevated.^1^ As such, other factors may be involved in the pathogenesis of atrial arrhythmias in these patients.

Atrial fibrosis is a key component of atrial structural remodelling in atrial fibrillation (AF) patients without congenital heart disease.^3^ Histological evidence of atrial fibrosis has been demonstrated in animal models of congestive heart disease,^4^ in the elderly^5^ and in patients with AF.^6^ Altered cellular conduction and disruption in cell-to-cell coupling occurring in the setting of fibrosis leads to the development of conduction velocity heterogeneity, conduction block, and increased vulnerability to the development and maintenance of AF.^7,^^8^

Atrial late gadolinium enhancement cardiac magnetic resonance (CMR) imaging has been used to quantify atrial fibrosis in non-congenital heart disease AF patients where the presence and extent of fibrosis are associated both with disease severity and recurrence of arrhythmia following AF ablation.^9,^^10^ Localized atrial fibrosis has been demonstrated on histological examination of left and right atrial appendage and free wall tissue sampled at surgical closure in one study of ASD patients.^11^ However, no studies have evaluated the presence of pan-atrial fibrosis, the relative burden of right and left atrial fibrosis, or the relationship between atrial fibrosis and arrhythmogenesis in this cohort.

We hypothesized that CMR-defined bi-atrial fibrosis is detectable in ASD patients and may be comparable in extent to that of non-congenital heart disease patients with paroxysmal AF. Given the preferential exposure of the right atrium to the haemodynamic consequences of the ASD from birth, we further hypothesized that (i) right atrial fibrosis may be of particular importance for arrhythmogenesis in ASD patients and (ii) progressive right atrial structural remodelling may be related to patient age and/or shunt size (Qp:Qs).

## Methods

This prospective cohort study conformed to the principles outlined in the Declaration of Helsinki. Ethical approval was granted by the Health Research Authority (17/LO/1218). Informed written consent was obtained prior to study participation. Consecutive adult patients >18 years with an uncorrected secundum ASD being considered for ASD closure were recruited to the ASD group. The comparison group consisted of adult patients with paroxysmal AF being considered for first-time AF ablation. Patients with significant co-existing structural heart disease including significant left ventricular hypertrophy, hypertrophic cardiomyopathy, and severe valvular disease were excluded. All patients underwent CMR imaging with dedicated bi-atrial late gadolinium enhancement sequences together with assessment of atrial area and volume. The presence of prior documented atrial arrhythmia on 12-lead ECG or 24-h continuous ambulatory monitoring was used to classify ASD patients into those with and without known atrial arrhythmias.

### Imaging protocol

All patients underwent CMR imaging on a 1.5 T scanner (Magnetom Area, Siemens Healthineers, Erlangen, Germany) using a previously described protocol.^12^ Short-axis imaging was performed through the atria using a standard bSSFP technique (slice thickness 8 mm, 50 phases). A respiratory navigated (acceptance window ±2.5 mm), ECG triggered, 3D whole heart magnetic resonance angiogram (MRA) was performed 90 seconds after infusion of 0.2 mmol/kg gadobutrol (Gadovist, Bayer Healthcare Pharmaceuticals, Berlin, Germany) with coverage to include both atria in axial orientation. For patients in sinus rhythm, scan acquisition was timed to atrial diastole, immediately prior to the opening of the mitral valve, with a duration of less than 150 ms to minimize atrial wall motion artefact. For patients scanned in atrial fibrillation, scan acquisition was similarly timed to the period following atrial filling immediately prior to mitral valve opening with the same constraint placed on acquisition duration (<150 ms). Twenty minutes after contrast administration, late gadolinium enhancement imaging was performed using an ECG-triggered, respiratory navigated, 3D whole heart, inversion recovery spoiled gradient echo sequence in axial orientation^13^ (spatial resolution 1.3 mm × 1.3 mm × 4 mm reconstructed to 1.3 mm × 1.3 mm × 2 mm, TR 4 ms, TE 2 ms, flip angle 20°), phase encoding direction; anterior–posterior, frequency encoding direction; right–left, parallel imaging; GRAPPA^14^ factor 2. A preceding single slice multi-phase inversion time mapping sequence (Look-Locker approach)^15^ was used to determine the correct inversion time to achieve adequate nulling of ventricular myocardium. In ASD patients, phase contrast imaging was performed in planes orthogonal to the aorta and main pulmonary artery to allow aortic and pulmonary flow calculation.

### Image processing

**Figure 1:**
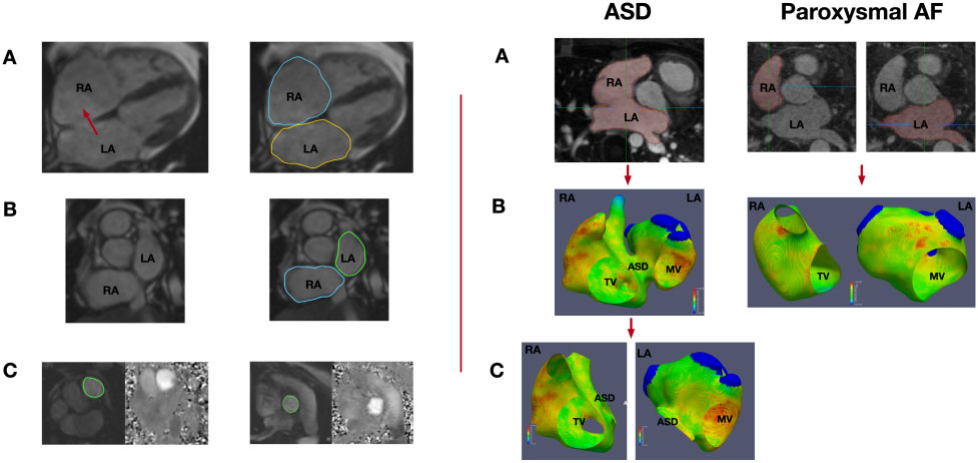
Left panel: Imaging processing steps. (*A*) Planimetry was used to assess right and left atrial area on four*-*chamber cine imaging at end atrial diastole. The red arrow indicates the location of the ASD. (*B*) Right and left atrial volumes were calculated from short-axis cine imaging. (*C*) Shunt fraction was calculated on phase contrast imaging of the pulmonary artery (left) and aorta (right). Right panel: Processing atrial fibrosis maps in ASD and paroxysmal AF patients. (*A*) Bi-atrial segmentations were created in ASD patients whereas the right and left atrium were segmented separately in AF patients. (*B*) Bi-atrial fibrosis map in an ASD patient and individual left and right atrial fibrosis maps in paroxysmal AF patient. (*C*) Individual right and left atrial maps generated in an ASD patient by clipping the bi-atrial map through the plane of the defect. LA, left atrium; RA, right atrium; MV, mitral valve; TV, tricuspid valve.

Right and left atrial fibrosis maps were created using CEMRG (cermgapp.com) according to previously published methods.^12,^^16^ Semi-automatic segmentations of the atrial blood pool were performed to create bi-atrial surface shells. In ASD patients, the right and left atria were segmented as one and clipped through the plane of the defect whereas separate segmentations of the right and left atria were created in non-congenital heart disease paroxysmal AF patients owing to the presence of the intact atrial septum. The pulmonary veins and left atrial appendage were removed from the left atrial shell and the inferior vena cava, superior vena cava, and right atrial appendage removed from the right atrial shell using clipping tools in CEMRG and Paraview (www.paraview.org) (*Figure 1*). Total atrial fibrosis burden was expressed as the percentage of the shell above a threshold of 1.2 times the mean signal intensity of the blood pool (IIR 1.2).^17^ Bi-atrial area and volume were measured using the CVI42 image analysis platform (CVI42, v5.1.1, Circle Cardiovascular Imaging, Calgary, ON, Canada). Right and left atrial areas were measured on four-chamber cine acquisitions at end atrial diastole. Right and left atrial volumes were calculated from cross-sectional slices generated from short-axis imaging through the atria. The shunt fraction (Qp:Qs) was calculated from phase contrast imaging of aortic and main pulmonary artery blood flow using CVI42 (*Figure 1***)** in ASD patients.

### Statistical analysis

Data analysis was performed using SPSS Statistics (IBM, Version 24) and Prism (GraphPad Software, Version 7). Normality of data was assessed using the Shapiro–Wilk test. Normally distributed continuous variables were expressed as mean ± standard deviation. Comparison of means between groups was performed using independent samples *t*-test for normally distributed data and Mann–Whitney *U* test for non-normally distributed data. Associations between continuous variables were assessed using Pearson’s correlation co-efficient for normally distributed data and Spearman’s correlation co-efficient for non-normally distributed data. An R-value of 0.0–0.39 indicated a weak relationship, a value of 0.4–0.59 a moderate relationship, and one of >0.6 a strong relationship.^18^ Receiver operating characteristic (ROC) analysis was used to determine fibrosis thresholds associated with atrial arrhythmia. Throughout, *P* < 0.05 was considered statistically significant. Since there is no prior data available on atrial LGE-CMR imaging in ASD patients a power calculation was not performed. Instead, consecutive patients were approached for participation from June 2017 to June 2019 and sample sizes of 36 patients per group were achieved.

## Results

### Baseline demographics

Thirty-six ASD patients (15 male, 21 female) and 36 non-congenital heart disease patients with paroxysmal AF (22 male, 14 female) were recruited (*Table 1*). The mean age was 50.4 ± 13.6 years in the ASD group vs. 60.3 ± 10.5 years in the paroxysmal AF group. The maximum defect size was 2.1 ± 0.7 cm and mean Qp:Qs was 2.2 ± 0.8. One ASD patient had a history of pulmonary hypertension. Eight ASD patients had a history of documented atrial arrhythmias. Four patients had persistent AF, two had paroxysmal AF and two had atrial flutter. Three patients in the ASD group and three patients in the AF group were scanned in AF with a controlled ventricular rate. All other patients were scanned in sinus rhythm.

**Table 1:** Baseline demographics of the study groups

	ASD group	AF group	*P*-value
Age	50.4 ± 13.6	60.3 ± 10.5	0.001
Male sex (*n*, %)	15 (41.7)	22 (61.1)	0.099
Hypertension (*n*, %)	7 (19.4)	11 (30.6)	0.276
Diabetes (*n*, %)	3 (8.3)	3 (8.3)	1.000
Stroke/TIA (*n*, %)	2 (5.6)	1 (2.8)	0.555
CCF (*n*, %)	0 (0)	0 (0)	
CAD (*n*, %)	1 (2.8)	7 (19.4)	0.024
CHA_2_DS_2-_VASc (mean)	1.1	1.5	0.421

AF, atrial fibrillation; ASD, atrial septal defect; CAD, coronary artery disease; CCF, congestive cardiac failure; CHA_2_DS_2-_VASc score, C, congestive cardiac failure, H, hypertension, A, age, D, diabetes mellitus, S, stroke or transient ischaemic attack, V, vascular disease, Sc, sex; TIA, transient ischaemic attack.

### Atrial area and volume

**Figure 2:**
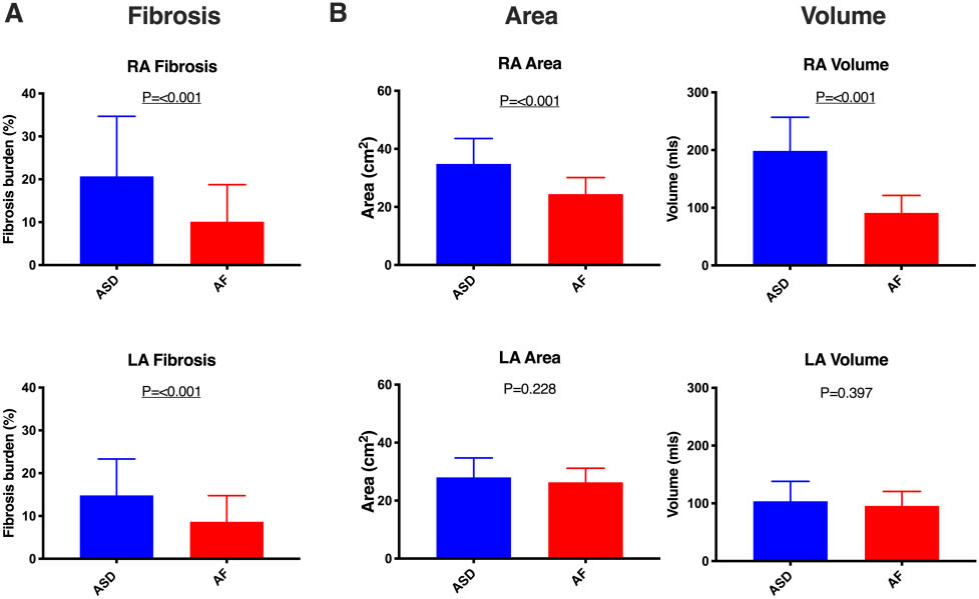
Comparison of atrial fibrosis (*A*) and atrial anatomical parameters (*B*) between ASD group and paroxysmal AF patients. Error bars represent mean ± standard deviation. LA, left atrium; RA, right atrium.

Atrial anatomical parameters are summarized in *Table 2*. Right atrial area and volume were both significantly greater in ASD patients than non-congenital heart disease AF patients (*P* < 0.001 for both), but there was no significant difference in left atrial area or volume between the study groups (*P* = 0.228 and *P* = 0.397, respectively) (*Figure 2*).

**Table 2:** Comparison of measured and calculated atrial structural parameters between the study groups

	ASD group	AF group	*P*-value
RA fibrosis (%)	20.7 ± 14	10.1 ± 8.6	<0.001
LA fibrosis (%)	14.8 ± 8.5	8.6 ± 6.1	<0.001
RA area, cm^2^ (cm^2^/m^2^)	34.8 ± 8.8 (17.7 ± 4.2)	24.4 ± 5.7 (11.9 ± 2.8)	<0.001
LA area, cm^2^ (cm^2^/m^2^)	28 ± 6.7 (14.1 ± 2.8)	26.3 ± 4.9 (12.9 ± 2.7)	0.288
RA volume, mL (mL/m^2^)	198.5 ± 58.3 (101.1 ± 28.5)	91.2 ± 30.2 (43.9 ± 12.9)	<0.001
LA volume mL (mL/m^2^)	103.7 ± 34.5 (51.4 ± 13.2)	95.5 ± 25.2 (46.5 ± 11.5)	0.397
RVEDV, mL (mL/m^2^)	273.3 ± 80.5 (143.5 ± 42.1)	159.9 ± 44.5 (78.3 ± 17.2)	<0.001
LVEDV, mL (mL/m^2^)	136.5 ± 41.6 (69 ± 17.7)	151.5 ± 41.4 (73.8 ± 15.6)	0.177
RVEF (%)	57.8 ± 8.9	58.2 ± 6.5	0.820
LVEF (%)	63.2 ± 8.3	61.3 ± 5.1	0.266

Atrial areas and volumes indexed to body surface area are provided in brackets.

LA, left atrium; LVEDV, left ventricular end-diastolic volume; LVEF, left ventricular ejection fraction; RA, right atrium; RVEDV, right ventricular end-diastolic volume; RVEF, right ventricular ejection fraction.

### Atrial fibrosis

**Figure 3:**
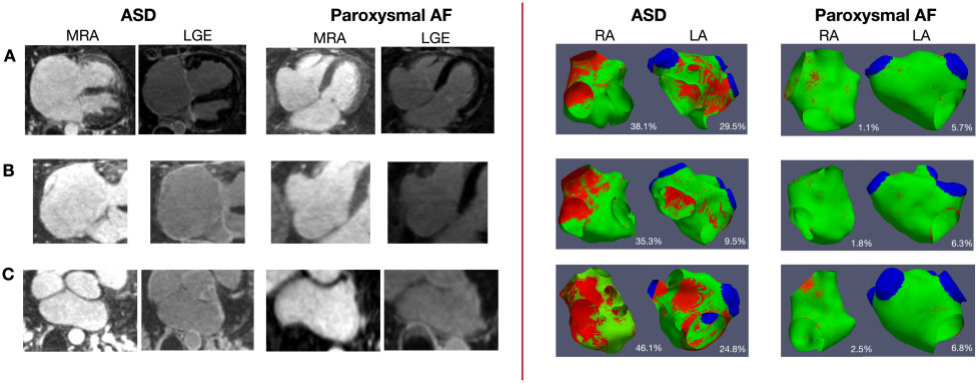
Left panel: Examples of MRA and LGE images generated in ASD and paroxysmal AF patients. (*A*) Four-chamber view of MRA and LGE images in an ASD and an AF patient. Right atrial enhancement can be appreciated in the ASD patient. (*B*) MRA and LGE sequences focused on the right atrium in an ASD and an AF patient with enhancement seen in the ASD patient. (*C*) MRA and LGE images focused on the left atrium in an ASD and an AF patient with enhancement seen at the posterior left atrial wall in the ASD patient. Right panel: Representative examples of right and left atrial fibrosis maps generated in ASD and paroxysmal AF patients. Red areas represent areas of detected late gadolinium enhancement. The fibrosis burden is stated as a percentage of the chamber area. Colour indicates signal intensity with red = above threshold, green = below threshold.

Atrial fibrosis maps were excluded from analysis in the case of poor image quality or if prior ablation had been performed on the ipsilateral side (one left atrial map in the ASD group and two right atrial maps in the paroxysmal AF group). Thirty-four right atrial and 33 left atrial fibrosis maps in the ASD group and 33 right atrial and 35 left atrial fibrosis maps in the paroxysmal AF group were therefore available for analysis. Representative late enhancement images and fibrosis maps are shown in *Figure 3*.

**Figure 4:**
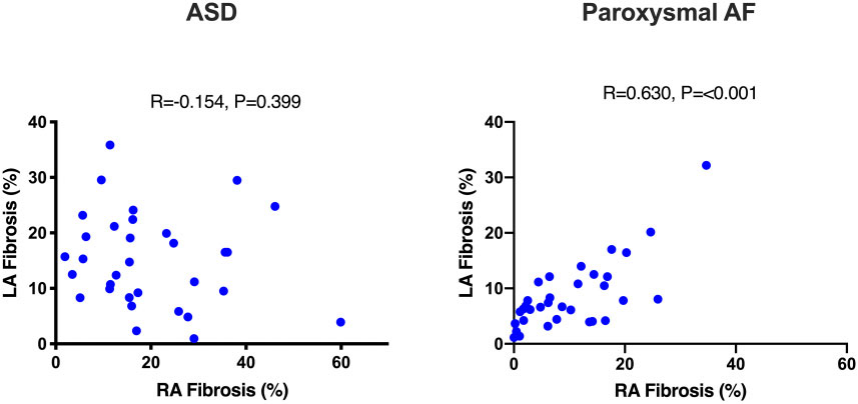
Scatter plots demonstrating relationship between right and left atrial fibrosis burden in ASD and paroxysmal AF patients. LA, left atrium; RA, right atrium.

Bi-atrial fibrosis burden was significantly greater in ASD patients than paroxysmal AF patients (right atrium, 20.7 ± 14% vs. 10.1 ± 8.6%; left atrium, 14.8 ± 8.5% vs. 8.6 ± 6.1%, *P* = 0.001 for both) (*Figure 2*, *Table 2*). In the ASD group, the mean fibrosis burden was greater in the right atrium than the left atrium although this difference did not reach statistical significance (20.7 ± 14% vs. 14.8 ± 8.5%, *P* = 0.116). Furthermore, no linear relationship was seen between left and right atrial fibrosis burden (*Figure 4*). In paroxysmal AF patients, fibrosis burden was similar in the right and left atria (10.1 ± 8.6% vs. 8.6 ± 6.1%, *P* = 0.209) with a linear relationship noted between the extent of fibrosis in each chamber (*R* = 0.630, *P* < 0.001, *Figure 4*). In the ASD group, right atrial fibrosis burden was associated with right atrial volume (*R* = 0.400, *P* = 0.032) but not with right atrial area. There was no association between left atrial fibrosis burden and left atrial area or volume in the ASD group.

### Association between atrial structural remodelling and atrial arrhythmias in ASD patients

**Figure 5:**
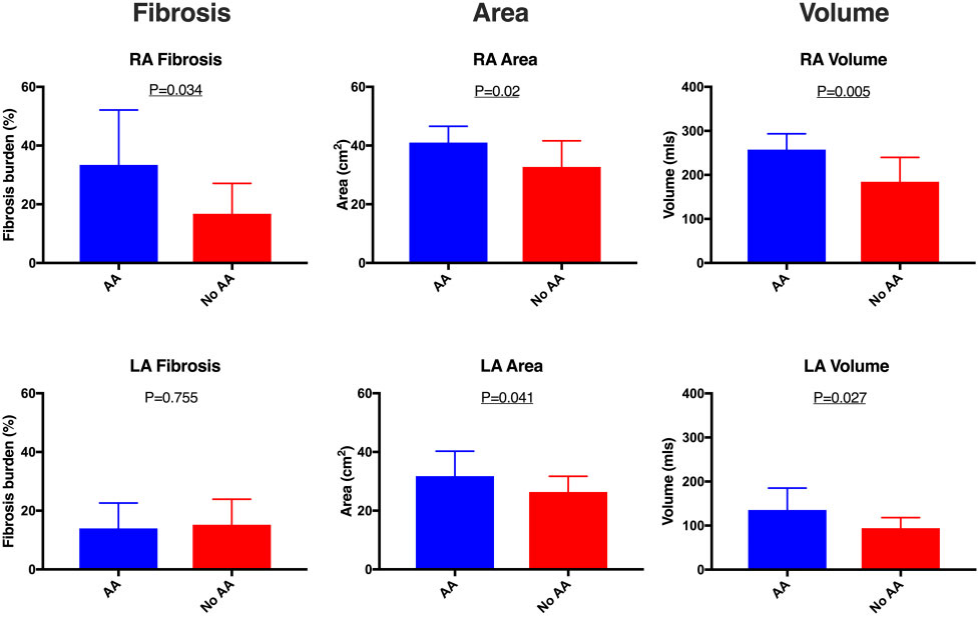
Barcharts demonstrating differences in measured and calculated parameters between ASD patients with (blue) and without (red) atrial arrhythmia. AA, atrial arrhythmia; LA, left atrium; RA, right atrium.

Patients with documented atrial arrhythmias were older than those without documented atrial arrhythmias (59.4 ± 10.3 years vs. 47.7 ± 13.7 years, *P* = 0.034). Right atrial area and volume were greater in ASD patients with, than without, atrial arrhythmias (41 ± 5.6 cm^2^ vs. 32.9 ± 8.8 cm^2^, *P* = 0.020 and 257.4 ± 35.9 mls vs. 184.6 ± 55.2, *P* = 0.005, respectively). Similarly, left atrial area and volume were associated with the presence of atrial arrhythmias (area, *P* = 0.041; volume, *P* = 0.027) (*Table 3*, *Figure 5*).

**Table 3:** Measured and calculated parameters in ASD patients with and without atrial arrhythmia

	ASD with AAs	ASD without AAs	*P*-value
RA fibrosis (%)	33.4 ± 18.7	16.8 ± 10.3	0.034
LA fibrosis (%)	14 ± 8.6	15.2 ± 8.7	0.755
RA area (cm^2^)	41 ± 5.6	32.9 ± 8.8	0.020
LA area (cm^2^)	31.8 ± 8.5	26.4 ± 5.3	0.041
RA volume (mL)	257.4 ± 35.9	184.6 ± 55.2	0.005
LA volume (mL)	135.5 ± 49.2	94.1 ± 24.1	0.027

AA, atrial arrhythmia; LA, left atrium; RA, right atrium.

**Figure 6:**
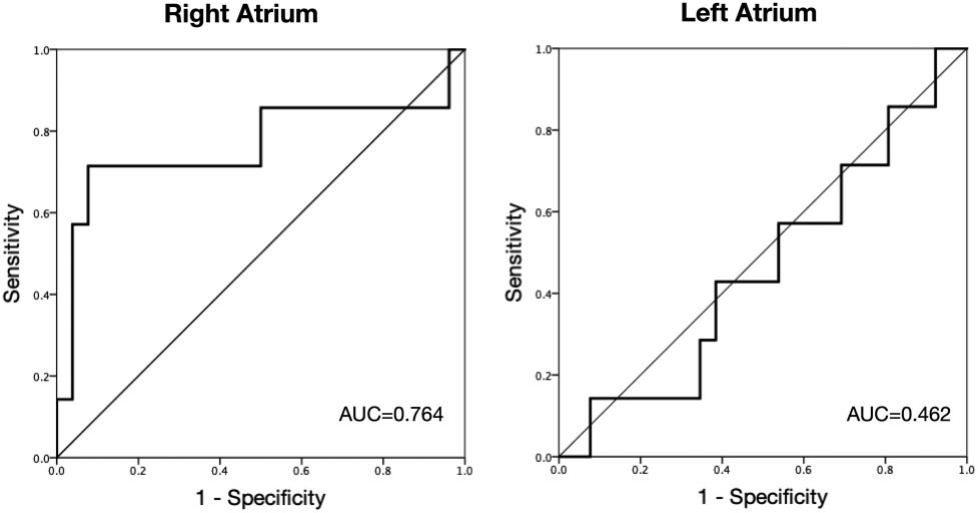
ROC analysis depicting sensitivity and one-specificity for right and left atrial fibrosis in the prediction of atrial arrhythmia in the ASD patients studied.

In the ASD group, right atrial fibrosis burden was significantly greater in those with prior atrial arrhythmias than those without (33.4% ± 18.7% vs. 16.8 ± 10.3%, *P* = 0.034, *Figure 5*). In contrast, no difference in left atrial fibrosis burden was seen in ASD patients with than without atrial arrhythmias (*P* = 0.755). Although not statistically significant, a more striking difference in the predominance of right relative to left atrial fibrosis was seen in those with arrhythmia (33.4 ± 18.7% vs. 14 ± 8.6%, *P* = 0.151) while those without arrhythmia had very similar levels of fibrosis in each atria (16.8 ± 10.3%, vs. 15.2 ± 8.7, *P* = 0.588). ROC analysis identified a right atrial fibrosis threshold of 32% as predictive of atrial arrhythmias with a 92% specificity and a 71% sensitivity (AUC 0.764) (*Figure 6*). No significant relationship was seen between the shunt fraction or defect size and the presence of arrhythmias (all *P* > 0.05).

### Association between age, shunt fraction, and atrial fibrosis in ASD patients

In the ASD group, a significant correlation was seen between age and right atrial area and volume (*R* = 0.430, *P* = 0.011 and *R* = 0.491, *P* = 0.004, respectively); however, there was no significant relationship between shunt fraction (Qp:Qs) and right atrial area or volume. Despite the association between right atrial fibrosis and atrial arrhythmia, neither age nor shunt fraction were associated with right or left atrial fibrosis in the ASD group (all *P* > 0.05).

## Discussion

The main findings of this study are (i) the extent of bi-atrial fibrosis is greater in patients with ASDs compared to non-congenital heart disease patients with AF; and (ii) right atrial, but not left atrial, fibrosis was associated with the presence of atrial arrhythmias in ASD patients. Despite the relationship between right atrial fibrosis and atrial arrhythmias, right atrial fibrosis was not associated with the duration or magnitude of the ASD shunt.

### The significance of bi-atrial fibrosis

To the best of our knowledge, this is the first study examining the presence and extent of bi-atrial fibrosis in ASD patients. We identified significantly greater bi-atrial fibrosis, with a predominance for the right atrium, in ASD patients compared to non-congenital heart disease patients with paroxysmal AF.

CMR-defined left atrial fibrosis is described in non-congenital heart disease AF patients and is associated with arrhythmia recurrence post-ablation.^10,^^19–21^ In contrast, very few studies have detailed the presence or extent of right atrial fibrosis in any patient population using late gadolinium enhancement imaging. In the largest series documenting right atrial fibrosis to date, a lesser degree of right compared to left atrial fibrosis was seen in 134 patients undergoing ablation^22^ with a linear relationship demonstrated between fibrosis burden in the right and left atria.^22^ Right atrial fibrosis has also been described in non-AF patients with structural or congenital heart disease. Two case series have described right atrial enhancement in patients with pulmonary hypertension and rheumatic heart disease.^23,^^24^ In a third study, right atrial enhancement was seen in 32.4% of patients with Ebstein’s anomaly which was additionally associated with the presence of supraventricular arrhythmia in these patients.^25^

In this study, right atrial fibrosis was significantly greater in ASD patients versus non-congenital heart disease patients with paroxysmal AF, highlighting the mechanistically distinct remodelling processes between these patient populations. Furthermore, in the ASD group studied here, no relationship was seen between the extent of right and left atrial fibrosis, however in paroxysmal AF patients, a linear relationship was noted between fibrosis burden in each atrium. In this study, right atrial fibrosis burden was also greater in ASD patients with atrial arrhythmias. As our comparison group consisted of patients with known AF, fibrotic remodelling was expected in this group. It is notable that the extent of bi-atrial fibrotic remodelling was greater in the ASD patients studied here despite a low prevalence of diagnosed atrial arrhythmias and a significantly younger age, highlighting the strong tendency toward the onset of atrial fibrosis in this population. The bi-atrial substrate in ASD patients is also emphasized by similar left atrial area and volume (*P* = 0.288 and *P* = 0.397) with respect to the ‘positive’ comparison AF group in whom left atrial dilatation is well described.

### Atrial fibrosis and arrhythmogenesis—clinical implications

No studies have systematically evaluated the contribution of right atrial fibrosis to arrhythmogenesis in any patient population. In the ASD cohort studied here, right atrial fibrosis burden was significantly greater in patients with a history of atrial arrhythmias than those without, however, left atrial fibrosis was not associated with atrial arrhythmogenesis in this cohort. These findings highlight a key difference in arrhythmia substrate between ASD patients and non-congenital heart disease patients with AF and may have important clinical implications for arrhythmia management strategies. While pulmonary vein isolation remains the treatment of choice for ASD patients presenting with AF, left-sided ablation alone may not be sufficient to treat atrial arrhythmias in many ASD patients with significant right-sided remodelling. Advanced right atrial structural and fibrotic change may underpin the pro-arrhythmic substrate in these patients, and a strategy of right-sided ablation in combination with pulmonary vein isolation alone may be of benefit and warrants future investigation.

The ROC analysis presented here identified a right atrial fibrosis burden of 32% as predictive of atrial arrhythmias in ASD patients with a high specificity. These findings could be useful in identifying ASD patients without manifest arrhythmia who are likely to develop, or already have undiagnosed, atrial arrhythmias. Such patients may benefit from rigorous rhythm monitoring and modification of any conventional reversible risk factors prior to ASD closure.

Increased bi-atrial volumes were associated with the presence of atrial arrhythmias in the ASD group. While prior studies have identified left atrial size as predictive of outcome and disease severity in non-congenital heart disease AF patients,^26^ a recently published study evaluating multiple indices of atrial remodelling using CMR identified only left atrial fibrosis as predictive of arrhythmia recurrence post-ablation on multi-variate analysis.^10^ Age was also associated with the likelihood of atrial arrhythmias in the present study, a finding which is consistent with prior studies of ASD patients undergoing closure.^27^ Age, however, represents a surrogate of structural remodelling rather than an accurate reflection of the specific arrhythmia substrate present in an individual patient. Based on the data presented here, right atrial fibrosis may represent a useful non-invasive clinical parameter for assessment of the risk of present or future atrial arrhythmias in ASD patients without manifest arrhythmia, regardless of age. These findings may also have implications for inter-atrial defects aside from secundum ASDs described here; however, the results presented cannot be extrapolated further without future studies exploring the interaction between atrial fibrosis and atrial arrhythmia in other specific patient groups.

The effect of ASD closure on atrial fibrotic remodelling has not been previously evaluated. Reduction in right atrial dimensions has been documented post-ASD closure, with chamber size pre-closure being a major predictor of remodelling potential.^28^ Chronic atrial stretch and dilatation stimulate fibrotic signalling pathways,^29^ and it is therefore possible that atrial fibrosis may reverse after the closure of the ASD. Conversely, the failure of reverse remodelling in patients with severe fibrosis on atrial LGE-CMR imaging may increase the future risk of atrial arrhythmias in such patients. Further studies are warranted to assess the interplay between atrial fibrosis and atrial arrhythmogenesis pre- and post-ASD closure.

### Right atrial fibrosis in ASD patients

In this study, we examined the relationship between age, shunt fraction and right atrial fibrosis to determine the effect of the duration and magnitude of the shunt on right atrial remodelling, but we did not show any significant relationship between these parameters. This may reflect the complex nature of fibrotic remodelling in these patients. In non-congenital heart disease patients the pathogenesis of atrial fibrosis remains incompletely understood with multiple fibrotic signalling pathways described at a cellular level.^30^ Furthermore, histological and CMR studies have suggested that age and cardiovascular co-morbidity are not always associated with fibrotic burden in AF patients.^20,^^31^ The present data therefore suggest that additional factors beyond age and the haemodynamic consequences of the shunt influence the development and progression of right atrial fibrosis in these patients.

Supporting the hypothesis that volume and pressure loading may contribute to the development of fibrosis, in patients with pulmonary hypertension, reduced right atrial voltage, as a surrogate marker for atrial fibrosis, has been demonstrated compared to normal heart control patients.^32^ Similar findings have also been seen in both atria in the setting of increased chamber dimensions and pressures secondary to rheumatic mitral stenosis.^33^ However, in non-congenital heart disease AF patients, a study examining CMR parameters associated with outcomes post-ablation reported only a weak association between left atrial dilatation and fibrosis burden suggesting that these processes may contribute independently to structural atrial remodelling and arrhythmogenesis.^34^ This is further supported by a histological study of surgical specimens which failed to show a relationship between atrial fibrosis and atrial dilatation in patients with ASDs with and without AF.^11^ In this study, a moderate linear relationship was seen between right atrial volume and fibrosis and between right atrial volume and age; however, no such correlation was seen between age and fibrosis, suggesting a lack of temporal relationship between the development of dilatation and fibrosis in this cohort.

Other potential contributory factors to the development of right atrial fibrosis may include genetic pre-disposition, environmental factors, and physical activity;^35,^^36^ however, their relationship to atrial fibrotic remodelling has yet to be explored in the ASD cohort.

### Limitations

Atrial LGE-CMR is subject to several inherent limitations and current imaging resolution can render the assessment of high signal intensity within the thin-walled atria challenging. Black blood imaging may improve blood pool to scar contrast however has yet to be systematically evaluated. Scan duration may be prolonged during AF which may affect myocardial nulling. For the purposes of this study, however, scans with evidence of incorrect nulling or motion artefact were excluded. The number of ASD patients with documented prior atrial arrhythmias presented here was small, and this study was not powered to detect differences between those with and without atrial arrhythmias. Prolonged ambulatory monitoring was not performed as routine in all patients therefore the true number of patients with atrial arrhythmias may have been under-estimated.

## Conclusion

Bi-atrial fibrotic remodelling is present to a greater extent in ASD patients compared to non-congenital heart disease patients with paroxysmal AF and right atrial fibrosis is associated with the presence of atrial arrhythmias in ASD patients. This study highlights the importance of the right atrium to arrhythmogenesis in ASD patients and the potential role of CMR imaging in the non-invasive assessment of these patients.

## Funding

This work was supported by the British Heart Foundation (FS/18/27/33543), the Wellcome EPSRC Centre for Medical Engineering at King’s College London (WT 203148/Z/16/Z), the National Institute for Health Research Biomedical Research Centre at Guy’s and St. Thomas’ NHS Foundation Trust and King’s College London and the National Institute for Health Research Cardiovascular MedTech Co-operative. S.E.W. is supported by the British Heart Foundation (FS/20/26/34952).

## Data Availability

The data underlying this article will be shared upon reasonable request to the corresponding author.
